# An ethnobiological study in Kala Chitta hills of Pothwar region, Pakistan: multinomial logit specification

**DOI:** 10.1186/1746-4269-10-13

**Published:** 2014-01-27

**Authors:** Muhammad Arshad, Mushtaq Ahmad, Ejaz Ahmed, Abdul Saboor, Azhar Abbas, Shumaila Sadiq

**Affiliations:** 1 Department of Botany, PMAS-Arid Agriculture University Rawalpindi, Rawalpindi, Pakistan; 2 Department of Plant Sciences, Quaid-i-Azam University Islamabad, Islamabad, Pakistan; 3 Department of Economics & Agricultural Economics, PMAS-Arid Agriculture University Rawalpindi, Rawalpindi, Pakistan

**Keywords:** Ethnobotany, Ethnozoology, Multinomial logit, Kala Chitta hills, Pothwar region, Pakistan

## Abstract

**Background:**

This paper constitutes an important ethnobiological survey in the context of utilizing biological resources by residents of Kala Chitta hills of Pothwar region, Pakistan. The fundamental aim of this research endeavour was to catalogue and analyse the indigenous knowledge of native community about plants and animals. The study is distinctive in the sense to explore both ethnobotanical and ethnozoological aspects of indigenous culture, and exhibits novelty, being based on empirical approach of Multinomial Logit Specifications (MLS) for examining ethnobotanical and ethnozoological uses of specific plants and animals.

**Methods:**

To document the ethnobiological knowledge, the survey was conducted during 2011–12 by employing a semi-structured questionnaire and thus 54 informants were interviewed. Plant and animal specimens were collected, photographed and properly identified. Distribution of plants and animals were explored by descriptive and graphical examination. MLS were further incorporated to identify the probability of occurrence of diversified utilization of plants and animals in multipurpose domains.

**Results:**

Traditional uses of 91 plant and 65 animal species were reported. Data analysis revealed more medicinal use of plants and animals than all other use categories. MLS findings are also in line with these proportional configurations. They reveal that medicinal and food consumption of underground and perennial plants was more as compared to aerial and annual categories of plants. Likewise, medicinal utilization of wild animals and domestic animals were more commonly observed as food items. However, invertebrates are more in the domain of medicinal and food utilization. Also carnivores are fairly common in the use of medicine while herbivores are in the category of food consumption.

**Conclusion:**

This study empirically scans a good chunk of ethnobiological knowledge and depicts its strong connection with indigenous traditions. It is important to make local residents beware of conservation status of species and authentication of this knowledge needs to be done in near future. Moreover, Statistically significant findings impart novelty in the existing literature in the field of ethnobiology. Future conservation, phytochemical and pharmacological studies are recommended on these identified plants and animals in order to use them in a more sustainable and effective way.

## Background

Evidences disclose the fact that human beings are familiar in the use of plants and animals for food, medicine, clothing etc. since ancient times [1]. Ethnobiology is the study of dynamic relationship among people, biota and the environment. More specifically, ethnobiology is the systematic domain that covers cultural study of how people learn, give name, use, and organize knowledge about the biota around them. Usually “Folk biology” is the term put to use by ethnobiologists to refer biological classification and reasoning specific to cultural groups [2]. Ethnobiology addressed big challenges to understand the perception and conceptualization of people about nature and health. It is also emphasizing the interaction of nature and health with socio-cultural, political and environmental conditions. Moreover, ethnobiology deals with the chances of change in political legislations as well as the attention of stakeholders in environmental and health concerns with free dissemination of science, knowledge and experiences [3].

Ethnobiology is a general term which is consisted with ethnobotany and ethnozoology as its key disciplines. The term ethnobotany was coined by J. W. Harshberger [4] as “the study of utilitarian relationship between human beings and vegetation in their environment, including medicinal uses”. A lot of work has been done on ethnobotany that has compiled the documentation of traditional ethnobotanical knowledge in most parts of the world including Pakistan [5-9].

Ethnozoology focuses on the relationship between animals and human beings for sake of food, medicine, art etc. It studies human practices of hunting, fishing and animal husbandry across space and time. Moreover, there are human practices about animals such as their place in the moral and spiritual realms [3]. A great variety of interactions between animals and human cultures are the subject matter of ethnozoology – “a science having deep roots within the human civilizations”. Human attitudes towards animals probably evolved long before current attempt to expose them artistically and scientifically [10]. It can also be elucidated that the origin of ethnozoology coincides with the appearance of human beings as specie [11]. There has been endeavoured a considerable work on ethnozoology in different parts of the world and a number of articles have been published online each year, but in Pakistan this discipline has been seldom explored [12-16].

The present research effort was carried out to present the ethnobiological facts from Kala Chitta hills located in the Pothwar region of Pakistan. Due to a combination of hills, plains and dynamic climate, it is rich in floral and faunal diversity. Therefore, this is considered a hotspot for biodiversity and ethnobiology. The people of the area cannot enjoy the fruits of modern facilities of civilizations due to lack of infrastructure and communication. The specific and distinguished socio-economic conditions of the region keep them closer to the natural resources. The area is rich in rural culture and folk traditions. People’s livelihoods are highly dependent on indigenous plants and animals. The importance of ethnobiology is reflected in their lifestyle including dressings, weddings, death ceremonies, childbirths, festivals, cultural functions and socio-religious beliefs. This area was not considered for the study of ethnobiological potential in the past for being far away from the main city and somehow prohibited by the Armed Forces. The present study is designed to document the traditional ethnobiological knowledge and association between ethnobotanical and ethnozoological facts. The inhabitants of Kala Chitta hills live in the area of great biological diversity that provides potent phytozootherapeutic remedies. People of this region have limited access to modern health facilities and public services. However, due to lack of money and the remoteness of the hilly range, plants and animals continue to play an important role in their daily life. The health services are based on use of medicinal plants and animals which is inexpensive and remedies are easily available. The historically close association between nature and locals of this Hilly range, almost all of the inhabitants have some rich knowledge about the use of medicinal plants and animals for treating a range of ailments.

This indigenous knowledge of medicinal plants has been put in danger currently due to the loss of traditional community life, widespread hunting of biodiversity and extensive use of fuel wood amidst deforestation. For this reason, this research endeavour sets out the first rigorous global study via multinomial logit specification to identify the probability of occurrence of diversified uses of plants and animals along with standard documentation of 91 plants and 64 animal species. The major goals of the present study are the documentation of ethnobiological knowledge regarding the utilization of medicinal plants and animals, the quantification of the data by applying analytical technique of Multinomial Logit Specifications (MLS) which is based on the number of positive responses for each species and the assessment of indigenous frequent uses of native species.

## Materials and methods

### Research area, climate, flora and fauna

**Figure 1:**
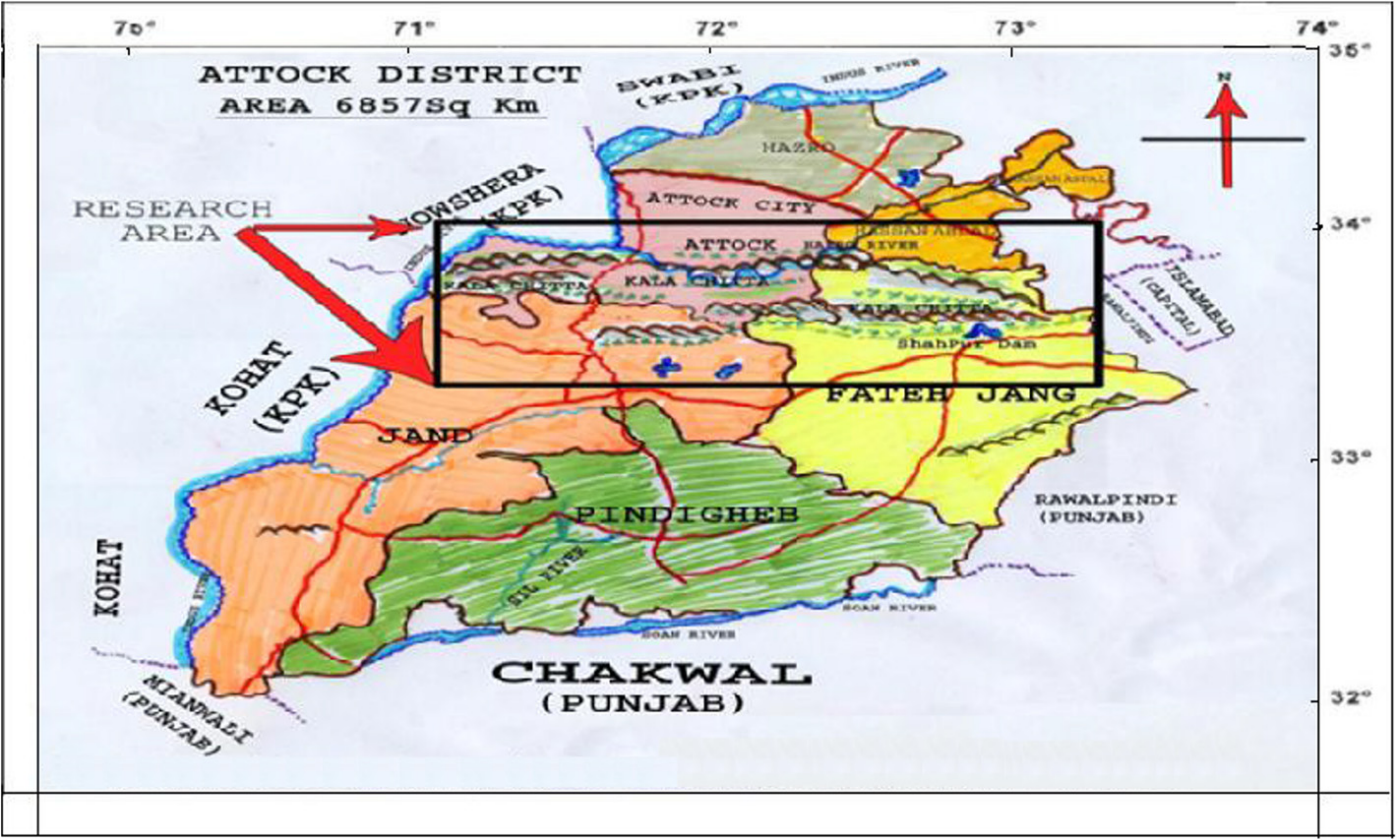
Map of the study area (Kala Chitta hills, District Attock, Pakistan).

Kala Chitta is a famous hilly range in district Attock which is located about 20 km North- West of Islamabad, the capital of the country. This area lies in North latitude between 33° 7’ and 34° while in East longitude between 71° 45’ and 73° (Figure 1). It is one of the largest hilly ranges in the Pothwar region of the Punjab province which runs across the northern part of the district and demarcates Attock from other districts. The hill appears to be a wedge with its base resting on the Indus River. It is gradually tapering eastward till it dies away on the border of the Fatehjang and Rawalpindi cities. The hills extend around 20 km in breadth and 77 km in length. These hills are naturally segmented into two parts. The South Western portion is known as “Kala Pathar” (Black Stone) and Northern side, “Chitta Pathar” (White Stone). They are having noticeable differences in appearance [17]. This area is inhabited by different tribes and clans namely *Pathan, Awan, Malik, Gujjar, Maliar, Syed, Sheikh* and *Mughals*. Most of the tribes in the area claim to be decedents of the invaders who migrated from Central Asia and Afghanistan [18].

Topographically, this area is a combination of hills and plains. The overall climate of study area is harsh with average minimum temperature of 17.92°C in January while 41°C average maximum temperature is observed in June. The rainfall pattern of the area is scanty and uncertain. The annual average rainfall is 605 mm per annum [19]. The soil comprises of soft grey sand-stones and orange to bright- red shale of the Siwalik system. In the north, there are several lime stone ridges which are coincided with Kala Chitta hills. The high ground on the North (near Attock and Lawrencepur) is formed by ancient rock series known as the Attock slates. These rocks are believed to be a great geological antiquity which may belong to the Precambrian system. Attock hills are formed of slates with veins of lime stones and whitish marble [20].

This area falls under dry temperate forests with vegetation and dominant species of *Acacia, Delbergia, Justacia, Dodonea, Olea* etc. Being a rich biodiversity centre, this area is also inhabited by a variety of wild animals. The most common animals are Leopard, Ravine Deer or Chinkara (Indian Gazelle or Hiran), Grey partridge (Tittar), Ordinary Bustard (Kharmohr) etc.

### Field exposure and ethnobiological data collection

The survey of the area was carried out between July, 2011 and June, 2012 to trace and document the ethnobiological knowledge. The ethnobiological research regarding data collection, plant collection and intellectual property rights (IPR) of local inhabitants were duly approved by the research ethics committee of National Biodiversity Action Plan for Pakistan and Herbarium situated in Quaid-i-Azam University, Islamabad. A method of semi-structured interviews was employed during the field survey to scan the ethnobotanical and ethnozoological information [21]. A total of 54 informants (32 male and 22 females) with different age groups were randomly selected for interviews. The selection of informants was mainly based on their rich indigenous knowledge and long term experience of utilization of plants and animals in the community. During the field surveys, general meetings, interviews of males and females, Herbal doctors/Physicians (Hakims) were conducted in addition to transect walks. Rural herbalists or Hakims were especially consulted for ethnobiological data and most of medicinal preparations were obtained from them.

Interviews based on semi-structured questionnaire were conducted with informants after explaining the aims of the study. Each questionnaire was divided into two parts including personal information data (name, age, education, occupation etc. and on plant and animal usages data (local names, traditional uses etc.). Plant samples were reported for medicinal uses and were photographed and collected. Thus herbarium specimens were prepared as per international standards [22]. The voucher numbers were assigned by the Herbarium (ISL) of Quaid-i-Azam University, Islamabad. The correct botanical labelling with author citation of the plants was reconfirmed by employing International Plant Names Index (IPNI) according to the standard rules of binomial nomenclature. After correct identification of the plants to be used using floral literature, the specimens were deposited in the Herbarium of Pir Mehr Ali Shah Arid Agriculture University, Rawalpindi, Pakistan for future research [23-26].

The identification of animals was completed by comparing with standard taxonomic keys and available literature [27-31]. Small animals like invertebrates were captured and after thorough identification, they were set free. Large animals were identified in the field as per respective folk description and subsequently with the aid of photo snaps.

### Methodological considerations for Multinomial Logit Specification (MLS)

Logistic regressions are the multiple regressions but used to predict a categorical outcome variable. They have two main specifications. First is the binary logit model specification which is used when the discrete response variable has two categories. Second is multinomial logit specification which is applied when the discrete response variables are multi-categorical [32-34]. Although binary logit model could be used in this study but multinomial logit specification was found to be more practical because of multiple uses of plants (and animals) and for its delivery of relatively more realistic findings.

The use of multinomial logistic models has gained prominence in predicting the relationship of plants and animals utilization with their respective categories. Multinomial logistic models dominate where the response variables are multi-categorized [33,34]. Individual has to choose only one alternative from the group of choices as (s) he is interested in how ceteris paribus changes in the elements of x which may affect the response probabilities, P(Y = j│x), j = 0, 1, …, J; since the probabilities must sum to unity. Multinomial logit model may be considered as a series of binary models where we evaluate the probability of the alternative j against alternative i for every i ≠ j. The specific multinomial logit formulation is adopted for the plants (and animals). The model so employed can be expressed as:

$$\ln\frac{P\left(Y=j\right)}{P\left(Y=0\right)}=\alpha_{m}+\sum_{k=1}^{J}\beta_{\mathrm{mk}}X_{\mathrm{ik}}+\mu_{i}$$

Where P(Y = j) is denoted by Pj and P(Y = 0) by P0;So logit becomes

$$\mathrm{Logi}t_{i}=\ln\left(\frac{P_{j}}{P_{o}}\right)=\alpha_{m}+\sum_{k=1}^{J}\beta_{\mathrm{mk}}X_{\mathrm{ik}}+\mu_{i}$$

In the study, the dependent variable includes different uses of plants (and animals) including medicinal and food (use together) and their all other usages (separate category). Plant categories are used as covariates in the analysis. They are part of “aerial and underground plant parts which include woody and non-woody plants” and “annual and perennial plants”. However, the animal categories used in the model consist of “domestic and wild animals”, “vertebrate and invertebrate animals” and “herbivore and carnivore animals”.

Multinomial logistic regression compares the multiple groups of “plants (animal) uses” through a combination of binary logistic regressions and estimates the number of equations less one category that is the base or reference category i.e. all other plants (animals) uses in this case. Reference group is normally selected with the highest numeric score that is the domain of “all other uses of plants”. Coefficients with the group’s reference are all zero just like that of binary logistic regression. Multinomial regression model does not impose the restrictions of normality, linearity and homogeneity of variances for explanatory variables. However, it follows maximum likelihood estimation and the chi-square distribution [32,35].

This logit is similar to logit in binary model [32,35] and interpretations of the logit estimates are also not very helpful in this case. However, the odd ratios and marginal effects are more useful in interpreting the relationships. The odd effects do not depend on the values of x but marginal effects clearly depend on x that can be seen from multinomial formulation of odd ratios and marginal effects [36].

## Results and discussion

### A profile of ethnobotanical inventory

**Figure 2:**
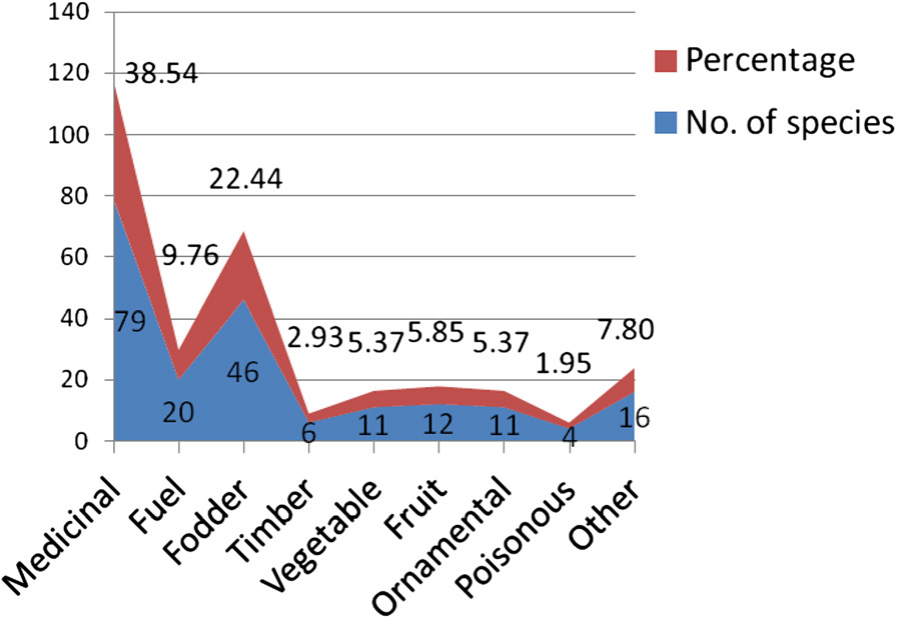
Ethnobotanical use categories (%) of plants of Kala Chitta hills, Pakistan.

During the survey, there were reported a total of 91 plants species belonging to 37 families and 79 genera which are being used ethnobotanically by the residents of selected area. The detailed inventory includes plant names (with local names), family, habit and ethnobotanical uses as provided in Table 1. Ethnobotanical analysis reveals that the reported species were predominately used for medicinal purposes (79 spp.) with 39%. It is followed by fodder (46 spp., 22%), fuel (20 spp., 10%), while rest of the categories usages are timber, vegetable, fruit, ornamental and poisonous etc. These were scarcely used (Figure 2). The ethnobotanical picture is reflected in Figure 2. There is clearly evident that the native species are well known by the indigenous people. These are endowed with rich heritage of ethnobotanical wisdom. They use different parts of plants as medicines, food, fodder, fuel, timber, and furniture etc. These people are dependent on these plant species to meet their daily life requirements. Qureshi and Bhatti reported that rural communities are more informative in terms of ethnobotanical knowledge than that of cities [37]. The reason is that this area is far from city and thus there is dearth of many basic needs. Many of the tribes are settled in this area since the time of Mughal emperors and Afghan invaders. Due to this reason, they are exposed to these plants generation after generations. They have great deal of experiences and wisdom regarding their use.

**Table 1:** Ethnobotanical uses of plants of Kala Chitta hills of Pothwar region, Pakistan

**S. no**	**Plant names/local name/herbarium specimen number**	**Family**	**Habit**	**Ethnobotanical uses**
01	*Abutilon indicum* (L.) Sweet*/*Peeli Boti/ISL-17	Malvaceae	Perennial herb	It is uses commonly as fuel wood and for grazing.
02	*Acacia modesta* Wall.//Phulai/Black wood/*Phulai*/ISL-19	Leguminosae	Moderate sized prickly deciduous tree.	Sweetmeat is formed from gum which is effective for lumbago. First of all, a quarter of kg of wheat starch is fried in equal quantity of animal fat for 10 minutes and then equal quantity of gum (which is already roasted to make soft) and sugar is added and again fried for 5–8 minutes. On cooling used for lumbago and as vigorous. Ash of bark is used along with mercury for paralysis, asthma and as aphrodisiac. Powder of dry bark along with little quantity of salt and sugar is used to treat chest pains and dysentery.
03	*Acacia nilotica* (L.) Delile/Indian Babul/*Kiker*/ISL-43	Leguminosae	A medium sized prickly tree.	The powder of dry pods and bark is effective for lumbago, kidney pains, diabetes, sexual disorders, and phlegm, as tooth powder and as astringent. Leaves decoction is used to treat dysentery. Gum is used in the formation of sweet meal which is effective for lumbago and recipe is same as mentioned in case of *Acacia modesta* Wall.
04	*Achyranthes aspera* L./Prickly chaff plant/*Puth kanda*/ISL-7	Amaranthaceae	Perennial herb	Ash of leaves and stem is recommended for piles, kidney stones, skin eruptions and asthma. Decoction of whole plant is used to treat pneumonia. Plant extract is used for dysentery and stomach-ulcer. Fried spines along with sugar are reported to be used in whooping cough by indigenous people.
05	*Aerva javanica* (Burm.F.) Juss ex Schult./Snow Bush/*Bui booti, Sufaid Bui*/ISL-49	Amaranthaceae	A small shrub	12 gram powder of dry leaves along with equal quantity of leaves of senna (*sana maki*), wild mint, nigella seeds (*kalongi*) and gugal (*gugul*) is ground and powder is used for epilepsy and insanity. (one table spoon twice a day). The same formula is ashed on coal and smoke is inhaled against insanity and epilepsy for 40 days.
06	*Albizia procera* (Roxb.) Benth./Chita Shirin/ISL-14	Leguminosae	Perennial tree	It is commonly used as fuel wood and shady tree.
07	*Albizia lebbeck* (L.) Benth./Siris/*Shirin*/ISL-15	Leguminosae	Large deciduous tree	Powder of dry seeds is effective for diabetes. Plant is antiseptic. Decoction of bark and seeds is effective for toothache and inflated gums, as astringent, piles and diarrhoea. Leaves extract is effective for skin diseases.
08	*Aloe barbadensis* Mill.*/Koar Gandal*/ISL-27	Asparagaceae	Perennial shrub	Mucilage of plant is used to improve skin. It is applied directly on skin.
09	*Aloe vera* (L.) Burm. f./Indian Aloe/*Kanwargandal*/ISL-28	Asparagaceae	Perennial succulent shrub.	Fresh jelly or cooked jelly with mutton is reported to be used for phlegm, diabetes, stomach ulcer, liver diseases. Jelly is astringent. Pickle of jelly is also recommended for diabetes. Powder of dry jelly along with bishop’s weed and black salt is used against constipation and as carminative. Jelly along with egg and curd is used as conditioner or hair tonic. Jelly along with honey and turmeric is applied on face as a mask for 15–20 minutes as cleanser, refresher and as skin tonic. Jelly along with 2–3 drops of lemon and rose extract is also used against freckles, pimples and boils.
10	*Amaranthus spinosus* L./Prickly amaranth/*Khardar chulari*/ISL-3	Amaranthaceae	Annual herb	Ash of plant is used to treat kidney stones (half tablespoon twice a day). Fresh leaves or leaves and stem are cooked as vegetable (*saag*) which is used to kill thread worms within body. Fresh leaves are cooked along with equal quantity of leaves of chicory plant (*kasni*) and fenugreek (*methi*) and used against low blood pressure and black cataract on eye (*kala motia*).
11	*Amaranthus viridis* L./Amaranth/*Chulai*/ISL-4	Amaranthaceae	Annual herb	Stem and leaves are cooked as vegetable and used against cough, inflammation and as urinative.
12	*Andrachne aspera* Spreng./Ramtutia/ISL-74	Phyllanthaceae	Annual herb	It is used for cough, bronchitis and dysentery.
13	*Anisomeles indica* (L.) Kuntze/Gulabi booti/ISL-37	Lamiaceae	Annual herb	Leaves are crushed and ground. Decoction of leaves is used to cure vomiting.
14	*Arachis hypogaea* L./Peanut/*Moong Phali*/ISL-61	Leguminosae	Annual herb	Fruit is used as caloric and vigorous. Oil is used in confectionery and ghee formation.
15	*Argyrolobium roseum* (Cambess.) Jaub. & Spach.*/*Makhni booti/ISL-75	Leguminosae	Annual herb	Aerial parts are used for tonic for sexual diseases.
16	*Asparagus adscendens* Roxb./Musli/ISL-45	Asparagaceae	Perennial herb	Rhizome powder is used for weakness of body and as sexual tonic
17	*Asphodelus tenuifolius* Cav./Bokhat/ISL-46	Liliaceae	Annual herb	Paste of the aerial part is used for skin allergy and skin infections.
18	*Avena fatua* L.*/*Jangli jontri/ISL-13	Poaceae	Annual herb	It is commonly used for live stock fodder.
19	*Azadirachta indica* A. Juss.*/Neem*/ISL-76	Meliaceae	Perennial tree	Leaves decoction is used at early morning for purification of blood.
20	*Boerhavia procumbens* Banks ex Roxb./Hog weed/It-sit/ISL-2	Nyctaginaceae	A prostrate spreading herb.	Garland is made by interlinking fresh slices of roots and put along neck of jaundiced person. With the improvement of disease length of garland is also increased. Paste of whole plant is used as antidote. Decoction of roots is used as refrigerant. The paste of fresh root along with equal quantity of cow ghee and henna is applied on hand and feet to relieve from irritation.
21	*Brassica nigra* (L.) K. Koch/Black mustard/Kali suron/ISL-6	Brassicaceae	Annual herb	Oil is conditioner, cleanser and skin tonic and caloric. For fair complexion local people dip its seeds in goat milk after noon prayer and night prayer they mesh seeds and applied on skin for 15–20 minutes as mask up to 11 days. Leaves and stem is cooked as vegetable (*saag*) and effective for phlegm, constipation, flatulence and used as diuretic.
22	*Bryophyllum pinnatum* (Lam.) Oken./*Zukhum-e-Hayat*/ISL-8	Crassulaceae	Perennial herb	Fresh leaves are used against kidney stones. Slightly fried leaves are used as astringent and dresser.
23	*Calotropis procera* (Aiton) Dryand/Swallow wort, Mudar,/Akk/ISL-5	Apocynaceae	Perennial shrub	Leaves and latex are used as antidote against snake and scorpion bite. Latex is also effective against wounds, and piles. 2–3 drops of latex is mixed in mustard oil and used as anti lice. Root paste and leaves as tissue paper is used externally for piles. 10 gm latex is mixed with 150 gram turmeric and pills are formed which are recommended for tuberculosis, Hepatitis B and C and spitting of blood (twice a day). Slightly fried leaves are used as dresser for inflated parts of body and rheumatism. 12 gm is heated along with 7 gm turmeric and 7 gm borax, when milk dried then removed from fire and pills are formed which are effective for cough, phlegm, as astringent, pain killer.
24	*Cannabis sativa* L./Indian hemp/*Bhang*/ISL-12	Cannabaceae	Annual erect herb.	Whole plant is intoxicant, laxative, narcotic. Ash of plant is used to treat bleeding at the nose and hemorrhoids. Ash along with date sweetmeat is vigorous and refrigerant.
25	*Capparis decidua* (Forssk.) Edgew./Karin/ISL-16	Capparaceae	Shrub	Aerial parts are used for fire wood and fruit is edible.
26	*Capsella bursa-pastoris (*L.) Medik./Shephard purse/ISL-18	Brassicaceae	Annual herb	It is commonly used for live stock fodder.
27	*Carrisa opaca* Stapf./Granda/ISL-11	Apocynaceae	Perennial shrub	Its fruits are edible and used for jaundice. Decoction of leaves is also used against jaundice.
28	*Carthamus oxycanthus* M. Bieb/Wild safflower/*Pohli*/ISL-10	Asteraceae	Leaves, Seeds	Roasted seeds or bread of its seed flour is used to treat jaundice, skin diseases, fever, scabies and abnormal eye sight and used as refrigerant. Decoction of leaves is used against dysentery. Local people give it to milk producing animals after delivery for two days and against dysentery.
29	*Carum coptium* (L.) Benth. & Hook. f./Ajwain/ISL-1	Apiaceae	Annual herb	It is used for digestive disorder, dyspepsia, vomiting and diarrhoea.
30	*Cassia angustifolia* M. Vahl./Negro coffee/*Kasundi*/ISL-9	Caesalpiniaceae	Perennial shrub.	Decoction of leaves along with pepper is effective for dropsy and cough. Diluted latex is used as eye drops which is effective for night blindness.
31	*Cenchrus ciliaris* L.*/Siti grass*/ISL-20	Poaceae	Annual herb	It is commonly used for live stock fodder.
32	*Chenopodium album* L./Goose foot plant/*Bathu*/ISL-22	Chenopodiaceae	Annual herb.	Decoction of leaves and stem is cooked as vegetable (*saag*) and used against tuberculosis, jaundice, blood purification, flue, phlegm, dropsy, diuretic, as caloric and against kidney and gall bladder stones.
33	*Cicer arietinum* L./Chickpea/*Kalay Cholay, Chunray*/ISL-26	Leguminosae	Annual herb.	Decoction of fruit, roasted fruit or turgid fruit is used to treat flue, cough, jaundice, diabetes, tuberculosis, phlegm, piles, kidney and liver diseases, blood purification etc. Flour of its pulse is mixed with milk, turmeric, lemon and mustard oil and ubtan is formed which is used as cleanser, skin tonic and refresher. Decoction of fruit along with honey is used to treat abnormal menses and throat pains. Bread made by its flour is also effective against diabetes.
34	*Cichorium intybus* L./Chicory/*Kasni*/ISL-24	Asteraceae	Leaves	Hadiths “There is chicory for you. On this plant dew drops of paradise fall every day”. Extract of leaves along with lemonade is used against chronic gastritis, and as liver tonic, diuretic, jaundice, dropsy.
35	*Citrullus colocynthis* (L.) Schrad./Bitter apple/*Tumma*/ISL-21	Cucurbitaceae	Annual creeping herb.	Fruit pulp along with almond oil and tragacanth is effective for rheumatism, paralysis, distortion of mouth, back ache and diabetes. Pulp along with sugar or honey is effective for chronic flue and sunstroke. Pickle of pulp is effective for diabetes. Fresh pulp along with black salt and bishop’s weed is given to animals when suffered from anorexia.
36	*Cleome brachycarpa* L./Wild mustard, Dog mustard/*Jangli gawara*/ISL-29	Cleomaceae	An erect, branched herb.	The herb is mixed with fodder and given to animals for enhancement of milk production.
37	*Convolvulus arvensis* L./Bind weed, Deer’s foot/*Lehli, Vehri*/ISL-23	Convolvulaceae	Climbing or twining herb.	Whole plant is cooked as vegetable (*Saag*) and used against skin diseases and as blood purifier. Decoction of whole plant is also used for the same purpose. Decoction of leaves is effective for constipation.
38	*Cucumus melo var. agrestis* Naudin/Chibar/ISL-30	Cucurbitaceae	Annual herb	It is used as wild vegetable, as digestive and against diarrhoea.
39	*Cymbopogon jwarancusa* (Jones) Schult./Wild lemon grass/ISL-25	Poaceae	A perennial grass.	Decoction of root is effective against dysentery. Powder of dry plant is used as astringent. The whole plant is vigorous so local people give it to animals as fodder to milk producing animals for more production of milk.
40	*Cynodon dactylon* L. Pers./Khabal grass/ISL-31	Poaceae	Perennial grass	Aerial parts are crushed and paste is applied on skin infection, injuries and eczema. It is also used as fodder.
41	*Cyperus rotundus* L.*/Dilla*/ISL-32	Cyperaceae	Annual herb	Rhizome of this plant is used to cure stomach diseases. It is crushed and ground to make powder. By mixing in water it is used thrice a day.
42	*Datura innoxia* Mill./Thorn apple/*Dhatura*/ISL-42	Solanaceae	An annual bushy herb.	Whole plant is intoxicant. Fresh or slightly fried leaves are used as dresser. Seed paste is effective for piles. Whole plant is burnt and smoke is inhaled for asthma. Local people pour fruit with cloves and kept it for 15 days. After 15 days they remove cloves and ground them with exact quantity of pepper and coconut and pills are formed which are used for tension, depression, urine problems, phlegm and during pregnancy for easy delivery.
43	*Dicanthium annulatum* (Forssk.) Stapf)/Murga Ghaa/ISL-51	Poaceae	Annual herb	It is used as fodder.
44	*Digera muricata* (L.) Mart.*/Jangli saag*/ISL-55	Amaranthaceae	Annual herb	It is a pot herb. It is used to cure skin diseases.
45	*Dodonaea viscosa* Jacq./Sanatha/ISL-73	Sapindaceae	Perennial shrub	Commonly used as fuel wood.
46	*Echinops echinatus* Roxb./Globe thistle/*Untkatara*/ISL-81	Asteraceae	Annual herb	Paste of root is applied on the belly of pregnant woman at child birth for easy delivery. Plant extract along with milk is used against anorexia, rheumatism and kidney stones.
47	*Eleusine indica* (L.) Gaertn./Barua/ISL-57	Poaceae	Annual herb	It is commonly used for live stock fodder.
48	*Eruca sativa* Mill./Rocket/*Tarameera, Jumian*/ISL-68	Brassicaceae	Annual herb	Oil is used as hair and skin tonic, effective for blood purification and against intestinal worms. Oil is boiled till it become sweet and given to animals as caloric. Seeds are effective for rheumatism (twice a day). 1 kg flour is boiled along with 5 kg of water, 1 kg of milk and 1 kg of sugar. When only 1 kg water is left then removed from fire and given to animals for more production of milk and to increase fat in milk.
49	*Eucalyptus globulus* Labill./Safaida/ISL-91	Myrtaceae	Tree	Commonly used for fuel wood, leaves are used for toothache and flue.
50	*Euphorbia helioscopia* L./Snake weed/Chatri Dudhak/ISL-71	Euphorbiaceae	Annual erect herb.	Milky latex is applied on cuts. Diluted latex is used as eye drops against eye problems.
51	*Euphorbia thymifolia* L./Prostrate spurge/*Tambi*/ISL-72	Euphorbiaceae	Annual herb	Plant extract is effective for piles. Decoction of plant is used for ring worms. Paste of plant is externally used against skin diseases.
52	*Fagonia indica* Burm.f./Fagonia/*Dhumian*/ISL-66	Zygophyllaceae	Perennial or annual spiny herb.	The extract of whole plant is antidiabetic, used against chicken pox, anticancerous, refrigerant and against skin diseases, scabies, toothache and blood purification.
53	*Ficus benghalensis* L./Banyan tree/*Bohr*/ISL-62	Moraceae	A large tree with stilt roots.	Latex from aerial parts is vigorous and used to fill cracking of feet. Milky latex along with little quantity of honey is used before fasting as antidiabetic. In case of sterility decoction of leaves, bark and root is used by local people up to two-three week.
54	*Ficus palmata* Forrssk./Wild fig/*Khabara, Angeer*/ISL-63	Moraceae	A deciduous tree.	Fruit is used for constipation, fair complexion, asthma, diabetes, flatulence, cough, liver diseases and inflammation. Fruit along with walnut is used as aphrodisiac, against kidney and gall stones, rheumatism, phlegm and piles. Latex of branches and powder of dry bark is used as toothpowder for toothache. Latex is used as massage for epilepsy and paralysis.
55	*Ficus religiosa* L./Sacred fig/*Peepal*/ISL-64	Moraceae	Large glabrous tree.	Fruit is laxative, astringent, refrigerant, used against asthma and constipation. Decoction of leaves is used for 40 days is a complete cure of gonorrhoea.
56	*Hordeum vulgare* L./Barley/*Joe*/ISL-54	Poaceae	An annual herb. Cultivated as cereal crop.	Fruit is roasted and powder is formed called barley flour (*Sattu*) which is dipped in water and next day after sieving and along with sugar is used against dysentery and as refrigerant. Bread of its flour is effective for blood purification, typhoid, heart diseases, as vigorous, and to fulfil iron deficiency. Fruit is mashed and cooked in milk along with honey and used against chronic constipation.
57	*Justicia adhatoda* L./Vasaka, Malabar nut/*Bhekar, Arusa, Bansa*/ISL-83	Acanthaceae	Perennial shrub	Leaves are insecticidal. Decoction of leaves is used to cure diabetes, and blood purification. Extract or powder of 25 gm dry leaves along with little quantity of honey or sugar is given during cough, asthma, spitting of blood and abnormal menses. Ash of whole plant is also effective for phlegm, cough and asthma by local people.
58	*Lallemantia royleana* (Benth.) Benth./Salvia seeds/*Tukhm-malanga*/ISL-47	Lamiaceae	A small erect herb.	Local people dipped seeds in water or milk in a clay pot and next day seeds with mucilage are used orally for dyspepsia, against high blood pressure, jaundice, as refrigerant and chest pain.
59	*Malva parviflora* L./Mallow/*Sonchal*/ISL-82	Malvaceae	Annual	Decoction of Leaves and stem or cooked as vegetable (*saag*) and used against phlegm, constipation and diabetes.
60	*Maytenus royleana* (Wall. ex M.A. Lawson) Cufod./Pataki/ISL-78	Celastraceae	Shrub	It is used as fuel wood.
61	*Melia azedarach* L./Barbadose lilac/*Dharek*/ISL-38	Meliaceae	A moderate sized tree.	Leaves extract is effective for leprosy, constipation, allergy, skin diseases, jaundice, piles, as astringent, and blood purifier. Leaves are boiled in 1 litre water when one-fourth water is left l then it is used for bathing against allergy and itching. One cup extract of leaves along with one cup extract of *Rhazya stricta* (verian) leaves is effective for diabetes before fasting. Leaves are given to animals as fodder in order to relieve them from inflammation.
62	*Mentha longifolia* (L.) L./Horse mint/*Pari poodna, chitta poodna*/ISL-41	Lamiaceae	An erect, aromatic herb	In case of dysentery local people used powder of its dry leaves along with black salt and bishp’s weed (one table spoon twice a day). Decoction of leaves is used for dysentery, colic pain, asthma, jaundice and stomach diseases.
63	*Mirabilis jalapa* L./Four o’clock plant/*Gul Basi*/ISL-79	Nyctaginaceae	Perennial herb.	The slightly fried leaves are used as dresser or ointment for wounds. The leaves are cooked as vegetable (*saag*) and used with bread against jaundice and dropsy. The powder of dry seeds is used to treat abnormal menses. Powder of dry flowers is effective for piles.
64	*Momordica dioica* Roxb. ex. Willd./*Jangli karaila*/ISL-88	Cucurbitaceae	Annual climbing herb	Fresh juice is recommended for diabetes. Powder of dry fruit along with dry leaves of *Peganum hermala* L. is recommended for jaundice.
65	*Morus alba* L./White Mulberry, *Chitta toot*/ISL-52	Moraceae	Perennial tree.	Fruit is vigorous. Fruit extract along with jam of quince seeds is used for sexual disorders and weakness for 25 days. The same formula is used for heart diseases and chest pain for 20 days.
66	*Morus nigra* L./Black Mulberry, *Kala toot*/ISL-53	Moraceae	Moderate sized tree	Fruits are edible, fruit extract along with water and sugar is used as tonic for cough, throat diseases including inflammation and tonsilitis.
67	*Olea ferruginea*(Sol.) Steud./Kahu, Wild olive/ISL-65	Oleaceae	Medium size tree.	Small leaves are used as herbal tea for cure of digestive complaints. Branches and trees are generally used for fuel wood, timber and preparation of agricultural tools. Cuttings of young stem are used as Miswak (Toothbrush).
68	*Opuntia monacantha* Haw/Prickly pear/*Thor, Nag phani*/ISL-89	Cactaceae	A xerophytic succulent shrub	Mucilage or ripe fruit is effective for gonorrhoea and syphilis. 4–6 drops of latex along with 10 drops of honey is effective for constipation. Ash of stem is also act as cathartic. Mucilage along with turmeric is externally used for piles, pox strains, rheumatism, and leprosy.
69	*Peganum harmala* L./Syrian tree, Wild rue/*Harmal*/ISL-90	Nitrariaceae	A perennial much branched bushy shrub.	The plant is used to drive away the evil spirits. Decoction of leaves is effective against intestinal worms, as aphrodisiac, and against backache. Seeds are decocted in olive oil and used to treat deafness. Fumigation of whole plant is used to treat toothache, chicken pox, and measles, to save from evil sprits and against sharply spreading diseases in plants, animals and human beings. Leaves are insecticidal. Seeds are intoxicant.
70	*Plantago ovata* Phill./Spogel seeds, Plantain seeds/*Ispaghol*/ISL-80	Plantaginaceae	Annual herb with rosette leaves.	Two tablespoon fruit bark of dried fruits dipped in one glass water, curd or milk and next day before fasting used it along with little sugar for dyspepsia. One table spoon of fruit bark along with 25 gram tragacanth and 12.5 gram basil seeds is effective for urine problems and as refrigerant.
71	*Polygonum plebeium* R.Br./Drunk/ISL-33	Polygonaceae	Annual herb	Aerial parts are used for jaundice, nose bleeding and diarrhea.
72	*Prosopis juliflora* (Sw.) DC./Mesquite/Kikri, Jand/ISL-44	Leguminosae	A Xerophytic shrub	Its honey is effective for kidney stones and kidney wounds. 12 gm its honey along with 12 gm kuthseereen and 12 gm wild mint is grinded and used (half table spoon twice a day) for freckles, asthma, cough, fair complexion and against boils and pimples. Branches are also used as tooth brush.
73	*Reptonia buxifolia* A. DC./Gunghair/ISL-48	Buxaceae	Shrub	It is used as fuel wood.
74	*Rhazya stricta* Decne/Rhazya/*Veriana*/ISL-50	Apocynaceae	Perennial shrub	The extract of leaves is used for skin diseases. Dry powder of leaves along with equal quantity of *Justicia adhatoda* leaves is used for indigestion. Its dry leaves along with equal quantity of chicory plant (*Kasn*i) and bishop’s weed (*Ajwain*) is mixed, grinded and used for menstruation problems, diabetes, and white cataract on eye.
75	*Ricinus communis* L./Castor oil plant/*Arand, harnoli*/ISL-34	Euphorbiaceae	An ever green shrub	Seeds are cathartic. Oil is used as massage for paralysis, as muscle tonic, inflammations. Root decoction or paste is used for piles. Leaves are used as dresser. Leaves decoction is effective for asthma and cough. Castor oil from its seeds is purgative.
76	*Rumex dentatus* L./Wild Spinach/*Jangli Palak*/ISL-35	Polygonaceae	Annual herb	Leaves and stem are cooked as vegetable (Saag) and used against flatulence. Root paste is used as astringent. Leaf paste is antiseptic.
77	*Salvia officinalis* L./Wild sage/*Gadkan*/ISL-36	Lamiaceae	A perennial herb.	Slightly fried leaves are used as astringent and as dresser. Plant is given to animals as vigorous.
78	*Saussurea heteromalla (*D. Don.) Hand.-Mazz*/Kali zeeri*/ISL-39	Asteraceae	Annual herb	It seeds are used for digestive complaints.
79	*Solanum nigrum* L./Black night shade/*Mako, Kanch manch*/ISL-40	Solanaceae	An annual herb.	Fresh leaves or leaves and stem after cooking are used for diabetes, abnormal eye sight, inflammation and hysteria. Fruit sauce is used as carminative.
80	*Solanum surattense* Burm.f./Wild egg Plant/*Mokri, Kandiari*/ISL-56	Solanaceae	A creeping perennial herb.	Fruit is given to animal for more production of milk and against anorexia. Decoction of leaves, stem and root is used as refrigerant. Powder of dry fruit is used as toothache against intestinal worms, stomach diseases, diabetes, boils and pimples. Fruit is warmed on spoon and fumigation is given against teeth worms.
81	*Tinospora cordifolia* (Willd.) Miers./Heart leaved moon seed/*Gillo*/ISL-58	Menispermaceae	A large climbering succulent shrub.	Decoction of fresh leaves and stem is used to kill worms present on the body of animals. Extract of leaves is used as astringent. Local people drink extract of 25 gm fresh leaves and stem along with 6 gm bishop’s weed (*Ajwain-i-desi*) and 12 gm salt in a clay pot having half liter water and kept it open whole day in sun light. Next day they mashed it well and used against chronic fever and malaria for one week.
82	*Tribulus terrestris* L*/*Small Caltrops/*Bukrha*/ISL-67	Zygophyllaceae	Annual herb	25 gram powder of dry fruit is ground along with 62 gram of bael fruit (*bael geri*) and used against dyspepsia, abnormal menses, as vigorous and as aphrodisiac. Powder of dry fruit along with milk is used for urine problems, gonorrhea, colic pains and leprosy. Very effective for kidney stones.
83	*Triticum aestivum* L./Wheat/*Kanak, Gandum*/ISL-59	Poaceae	An annual herb. Cultivated as cereal crop.	Bread of flour is used to treat inflammations, diabetes and piles. Bread is also effective for pregnant women, and as caloric. Fruit and stem is vigorous so given to poultry for more production of eggs and meat and milk producing animals for more production of milk. Wheat fibres are dipped in water and next day after sieving used for diabetes by local people. Local people made a special food called sureed by cooking wheat meal with meat and milk and used it as aphrodisiac, vigorous and against sexual disorders. Wheat starch is used in the formation of sweetmeat which is effective for lumbago. Bread of unsieved flour of wheat is effective for constipation.
84	*Verbascum Thapsus* L.*/*gidar tambaco/ISL-70	Scrophulariaceae	Perennial herb	The whole plant is dried, powdered and is use to ease menstrual flow, relieve constipation and its high dose is used in abortion.
85	*Viola stocksii* Boiss*.*/Makhan Boti/ISL-*69*	Violaceae	Annual herb	The leaves paste is mixed with water and brown sugar and is given orally against diarrhoea and dysentery.
86	*Vitex negundo* L./*Marvan*/ISL-60	Lamiaceae	Shrub.	Leaves paste is used against rheumatic and joint pain.
87	*Wattakaka volubilis* (L. f.) Stapf./ISL-84	Asclepidaceae	Perennial shrub	The leaves past is mixed with brown sugar to be used against cough, cold and other respiratory problems.
88	*Withania coagulans* (Stocks.) Dunal/Withania/*Paneer dodi, Ashwagandha*/ISL-87	Solanaceae	Perennial shrub	Decoction of leaves is used orally in very small amounts, and externally for taking bath against skin diseases. Leaves and branches are placed in stored wheat grains and other cereals to avoid insect pests. Dried branches are also used for fuel.
89	*Withania somnifera* (L.) Dunal/Winter Cherry/*Aksin, Asghand*/ISL-86	Solanaceae	A small perennial erect shrub.	Flowers are dried and powdered. This powder is used locally by females for abortion. These are also used in fewer amounts to ease menstrual flow. Leaves are used as fodder and Branches as fuel.
90	*Zizyphus mauritiana* Lam.*/*Bairi/ISL-85	Rhamnaceae	Tree	Fruits are edible, leaves are used for diabetes and hair tonic.
91	*Zizyphus nummularia* (Burm.f) Wight and Arn/Wild jujube/*Jahri Ber, Beri*/ISL-77	Rhamnaceae	Tree	Powder of dry fruit is effective against vomiting and given to women before pregnancy. Poultice of fresh leaves along with soap and flax leaves are used as dresser and astringent. 1/4 kg fresh leaves of (*beri)* along with 1/4 kg leaves of *Amla*, 12 gm (*sika kai*) and 1/8 Kg of soap nut (*Reetha*) are boiled in 4 kg water and used as hair tonic.

**Figure 3:**
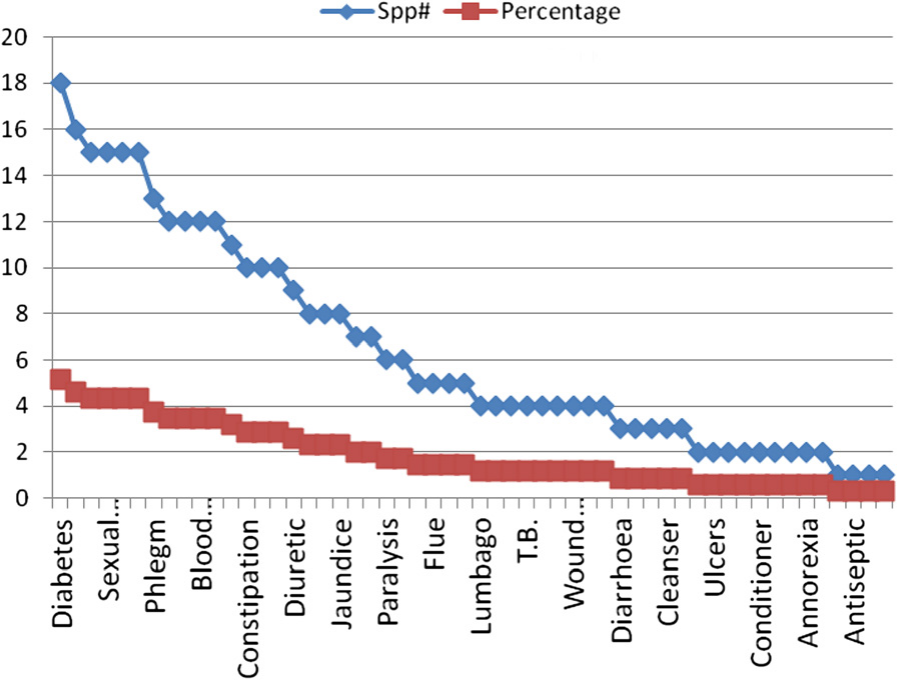
Diseases (%) treated by number of plant species.

**Figure 4:**
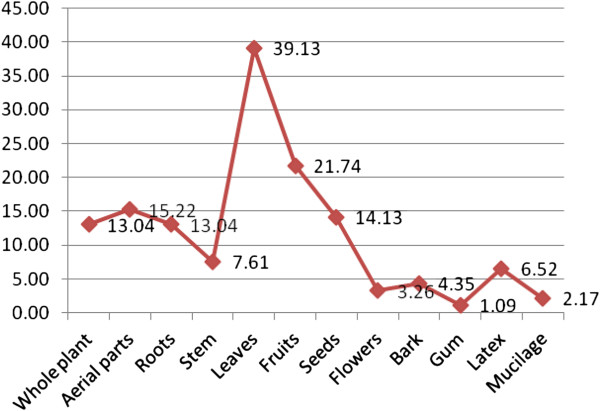
Part used (%) of plants of Kala Chitta hills, Pakistan.

The plant species used for the treatment of a particular disease is indicated in Figure 3. It shows that maximum numbers of plant species (i.e. 18) are used for the treatment of diabetes and as pain killer (16). However, there are a few plants which are used for the treatment of pneumonia and skin diseases (Figure 3). Figure 4 shows the percentage of the plant’s parts being used for ethnobotanical purposes. The maximum usage of ethnobotanical species is recorded of leaves (39%), fruits (22%), aerial parts (15%), seeds (14%), and roots (13%). As in this study, the people of Kotli Sattian do possess similar kinds of plant’s parts which are used for making recipes [38].

The inventory of economic plants is shown in Table 1. It reveals that people of remote areas are still dependent on plants. However, the urban people are not familiar with most of these plant usages especially that of medicinal ones. It is dire need of the time to fill this knowledge gap and document the entire information so that it can be preserved and used for future research and verification. In spite of all these awareness, the native inhabitants are still ignorant of the importance of biodiversity and its conservation. It is highly important to educate them so that loss of plants may be minimized. There is call for more comprehensive surveys and study projects to make people aware about the need of documentation of the entire knowledge and explore their importance to the natives [38].

### A profile of ethnozoological inventory

Kala Chitta range has an immense grazing potential in addition to a number of wild species of animals including *Amphisema stolatum, Apis mellifera, Helogale parvula-herpestes edwardsi, Hypomelas micrpus, Laepus nigricollis, Melanoperdix niger, Naemorhedus goral, Oryctolagus cuniculus, Rhesus macaque, and Uromastyix hardwicki* etc. Most of these are common in this area. Moreover, livestock is an additional source of income for most of the people because they are not economically well off. There is also hunting pressure on wildlife species (including *Oryctolagus cuniculus, Melanoperdix niger, and Naemorhedus goral*) due to lack of law enforcement and conservation concept. These are at the risk of destruction danger in the study area.

**Figure 5:**
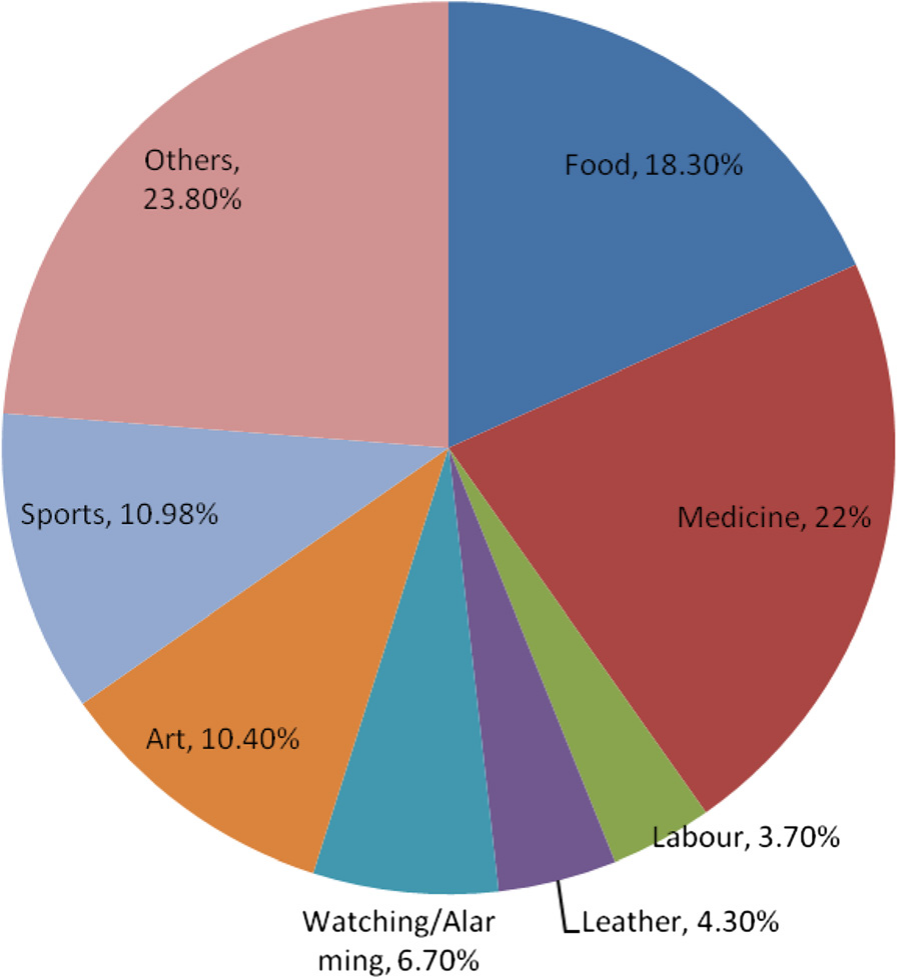
Use categories (%) of animals of Kala Chitta hills, Pakistan.

Around 64 animal species belonging to 45 families and 62 genera were explored from the study area. The detailed ethnozoological inventory of animals is profiled from this area which reveals that local people have versatile consumption of these animal species (Table 2). The percentage analysis of these ethnozoological inventory shows that maximum number of animals are being used for the medicinal purpose (22%), along with food (18%), sports (11%) and art (10%) purposes (In Figure 5). Though this area is enriched in ethnozoological wisdom but this unique knowledge is available with the elders only. The young generation is not much familiar and concerned with the facts of ethnozoological spectrum of the region due to cultural changes. Hence this deteriorating information should necessarily be preserved by means of identification and documentation.

**Table 2:** Ethnozoological uses of animals of Kala Chitta hills of Pothwar region, Pakistan

**S. no**	**Animal names/local name**	**Family/class**	**Phylum**	**Ethnozoological uses**
01	***Acridotheres gigninianus***/Kattar, Bank Myna.	Sturnidae/Aves	Chordata	It is captured and used as pet. It is trained and used to speak like parrot. It is also used as alarming bird against snakes, mongoose, jackal etc.
02	***Acridotheres tristis***/Kattar, Common Myna	Sturnidae/Aves	Chordata	It is captured and used as pet. It is trained and used to speak like parrot. It is also used as alarming bird against snakes, mongoose, jackal etc.
03	***Alectoris chukar***/Chakor	Phasianidae/Aves	Chordata	Hunted for meat and for fun. Stuffed chakor is also used for ornamentation. It is also used as sports bird for partridge fight.
04	***Amphisema stolatum***	Coloubridae/Reptilia	Chordata	Used as pet by jugglers.
05	***Anas platyrhynchos***/Batukh	Anatidae/Aves	Chordata	Kept in houses and used for meat and eggs.
06	***Apis mellifera****/Shehd di makhi*	Drosiphilidae/Insecta	Arthropoda	Honey is not only used as food but also used for a number of medicinal values. Wax is used for making candles and articles for ornamentation. Empty honey comb is also used in interior decoration.
07	***Bos tarus***/Gaan, cow	Bovidae/Mammalia	Chordata	Used as pet for milk and meat. Milk is also used to make curd, cheese, butter and deesi ghee. They are also used in ploughing fields, in Raahatt for water-well, in carts etc. Bulls are used in bull race. Dung is used as fertilizer. Hides are used in leather industry.
08	***Bubalus bubalis***/Buffalo, Munj, Bheins	Bovidae/Mammalia	Chordata	Used as pet for milk and meat. Milk is also used to make curd, cheese, butter and deesi ghee. Hides are used in leather industry. Dung in used as fertilizer. Curved hornes are used as decoration article. Bones are used in making utensil handles.
09	***Bunglaris spp***/Sang choor, Krait	Elapidae/Reptilia	Chordata	Deadliest poisonous snake, its venome is used for production of anti-venome.
10	***Calotes versicolor***/Rat-mundia	Agamidae/Reptilia	Chordata	Often hunted and kept by jugglers to attract people.
11	***Camelus dromedaries***/Oont, Dachi, Camel	Camelidae/Mammalia	Chordata	Used as pet animal for carriage and also used for meat. Its meat is salty. Its milk is used for the treatment of Hepatitis B and C.
12	***Canis lupus familiaris***/Kutta, Dog	Canidae/Mammalia	Chordata	Used as pet animal for dog fight, dog race, for monitoring and alarming at home.
13	***Capra hircus***/Bakri, Goat	Bovidae/Mammalia	Chordata	Used as cattle for milk and meat. Its hide is used in making leather articles. Its milk is preferred for infants.
14	***Catla Catla***/Machli, Thaila	Cyrinidae/Actinopteriygii	Chordata	Used as food after cooking. Its oil is used in joint pain. Scales are used in paintings with glue and paint spray.
15	***Chana marulius***/Sap machli, Saul	Chanidae/Osteichthyes	Chordata	Used as food. Its oil is used in joint pain.
16	***Cirrhinus mrigala***/Mori	Cyprinidae/Actinopterygii	Chordata	Used as food after cooking. Scales are used in paintings with glue and paint spray. Its oil is also used in joint pain.
17	***Clupisoma naziri***/Bachwa	Shilbeidae/	Chordata	Used as food after cooking. Its oil is used in joint pain. Scales are used in paintings with glue and paint spray.
18	***Columba livia***/Kabootar, Pigeon	Columbidae/Aves	Chordata	Kept as pet birds and used for meat. Its is also given to patient for treatment of paralysis. It is also used for early onset of puberty in young girls. Its a sports bird and used in pigeon race. Its feathers are used in decoration articles, dolls and in shuttle cocks.
19	***Corvus splendens***/Kaan, Kawwa	Corvidae/Aves	Chordata	Presence of this bird at home is said to be an indication for arrival of some guests.
20	***Cyprinius carpia***/Gulfam	Cyprinidae/	Chordata	Used as food after cooking. Its oil is used in joint pain. Scales are used in paintings with glue and paint spray.
21	***Danus plexipus****/*Butterfly, Tittlee	Nymphalidae/Insecta	Arthropoda	Preserved forms are used in horticulture.
22	***Dendrocitta vagabunda***/Kamadi Kukkarh	Corvidae/Aves	Chordata	Kept as pet and used for meat.
23	***Echis carinatus***/ghoona, krait	Viperidae/Reptilia	Chordata	Captured by some people to sell to snake charmers or to bio-medical laboratories for anti-venome production.
24	***Equs asinus Xe. Cabalus***/Khachar, Mule	Equidae	Chordata	Uses for carriage purpose.
25	***Equs caballus***/Horse, Gorha	Equidae/Mammalia	Chordata	It is used as pet animal. It is used for carriage, horse-race, naiza-bazi etc.
26	***Equus asinus***/Khoota, Gaddha, Donkey	Equidae/Mammalia	Chordata	Kept as pet animal and used for carriage purpose.
27	***Eryx johani***/Domoi	Boidae/Reptilia	Chordata	Biological control for Mus musculus etc. Which destroy crops.
28	***Felis silverstris ornate***/Cat, Billi	Felidae/Mammalia	Chordata	Kept as pet and controls mouse and lizards.
29	***Gallaus gallus domestica***/Kukkar, Kukri	Phasianidae/Aves	Chordata	Kept at home for meat and eggs. Its meat is cooked in various forms.
30	***Germen eve***/Sheep, Bhairh	Bouidugs-caprinae/Mammalia	Chordata	Kept as pet and used for milk, meat and wool. Its hide is used in making leather articles. It is also sacrificed by muslims at Eid-ul-Azha.
31	***Hamidactylus flavividus***/Chipkli, Lizard	Gekkonidae/Reptilia	Chordata	Biological controller for insects, cockroach, flies, mosquitoes etc.
32	***Haplobatrachus tigerinus***/Mendak, Frog, Duddu	Ranidae/Amphibia	Chordata	Used in dissection experiments, also captured and used for snake diet.
33	***Helis aspera***/Seepi	Helicidae/Gastropoda	Mollusca	Shells are used as ornamental after painting.
34	***Helogale parvula-herpestes edwardsi***/Mongoose, Neola	Herpestidae/Mammalia	Chordata	Some people it to drive away snakes. Jugglers also keep it for fight with snakes.
35	***Homo sapien/*** Insan, Banda	Hominidae/Mammalia	Chordata	It is highly social animal which is used in art, culture, as labour and in many other professions from social welfare to life saving.
36	***Hypomelas micrpus***/Hedghog, chayya	Erinaceidae/Mammalia	Chordata	It is kept in houses to drive away snakes, lizards and mouse.
37	***Labeo rohita***/Rohu, machli	Cyprinidae/	Chordata	Used as food after cooking. Its oil is used in joint pain. Scales are used in paintings with glue and paint spray.
38	***Laepus nigricollis***/Sayya, Khargosh	Leporidae/Mammalia	Chordata	It is hunted for its meat, which is also used for treatment of bronchial diseases. Its fur is used in wallets, warms caps etc.
39	***Lumbricus terrestris***/Ketchwa, Earthworm	Lumbricidae/Polychaetae	Annelida	It is used to capture fish.
40	***Mabyua carinata***/Fatmi guddi, Skink	Scindidae/Reptilia	Chordata	Some people believe it as noble reptile, and kept it as pet for short time because it cannot survive long captivity.
41	***Melanoperdix niger***/Black partridge, Kala Teetar	Phasianidae/Aves	Chordata	Kept as pet-bird. Used for meat and its soup is used to treat bronchitis. It is used in games for prolonged singing voice competition. It is also used to attract other wild partridges for capturing.
42	***Milvus migrans***/Eill, Cheel	Accipitriformes/Aves	Chordata	Sold for zoo and wildlife sanctuaries.
43	***Mystus singhala***/Singhare	Bagridae/Actinopterygii	Chordata	Used as food after cooking. Its oil is used in joint pain. Scales are used in paintings with glue and paint spray.
44	***Naemorhedus goral***/Deer, Hiran	Bouidugs/Mammalia	Chordata	It is hunted for its meat, which is also used in treatment of asthma. After stuffing it is used in ornamentation and in scientific labs.
45	***Naja naja***/Naag, chajlap, Cobra	Elapidae/Reptilia	Chordata	Fat of cobra is used in muscular pain, sexual weakness and arthritis. It is also captured and sold to laboratories for anti-venome production.
46	***Oenanthe fusca***/Pahari chirili	Muscicapidae/Aves	Chordata	Its faeces are used in constipation and other gastric problems in infants.
47	***Oryctolagus cuniculus***/Rabbit, Sayya, Khargosh	Leporidae/Mammalia	Chordata	It is hunted for its meat, which is also used for treatment of bronchial diseases. Its fur is used in wallets, warms caps etc.
48	***Pachydondyla vernae***/Ant	Formicidae/Insecta	Arthropoda	Fine mud of their houses is mixed with water to make a paste, which is applied on mumps affected area to get relief.
49	***Passer domesticus***/Chirhi	Passeridae/Aves	Chordata	Meat is used as food. Faeces paste is used orally in gas trouble and constipation.
50	***Perdix perdix***/Bhoora Teetar, Grey Teetar	Phasianidae/Aves	Chordata	Used as pet-bird and for meat.
51	***Photuris lucicrescens***/Firefly, Jugnu	Lampyridae/Insecta	Arthropoda	Children capture them and used to play them because of their lightening.
52	***Psittacula krameri***/Mian Methoo, Toota. Parrot	Psittaculidae/Aves	Chordata	It is used as pet-bird, as it can speak few words like humans after trained. Many palmists use it to highlight their business as lottery chit or fate-fortune.
53	***Pycnonotus cafer***/Bulbul, Nightingale	Pycnonotidae/Aves	Chordata	Kept as pet-birds due to its singing habit, also used in painting.
54	***Reticuliternes banyulensis***/Seekh, Termite or White ant	Termitidae/Insecta	Arthropoda	Fine mud of their houses is collected and used for carving writing on walls with sharp objects, or claying writing.
55	***Rhesus macaque***/Monkey, Bandar, Bhuja	Circopithecoidae/Mammalia	Chordata	These are captured by jugglers and trained to amuse mob to earn money.
56	***Rita rita***/Khagga	Bagridae/Actinopterygii	Chordata	Used as food after cooking. Its oil is used in joint pain. Scales are used in paintings with glue and paint spray.
57	***Streptopelia deceocto***/Kughi, Faakhta	Columbidae/Aves	Chordata	It is often hunted for its meat, which is used to treat cough and for early onset of puberty in girls. Its feathers are used in decorations and in shuttle cock.
58	***Sus sucrofa***/Pig, Soor, juhi wala	Suidae/Mammalia	Chordata	It digs fields at night and makes them fertile by their faeces but destroys crops alot.
59	***Tor marcolepis****/Mahasheer*	Cyprinidae/Teleostomi	Chordata	Used as food after cooking. Its oil is used in joint pain. Scales are used in paintings with glue and paint spray.
60	***Upupa epops***/Hud-hud, Tarkhan	Upupidae/Aves	Chordata	Feathers are used in art work and in making dolls.
61	***Uromastyix hardwicki***/Saanda	Aganidae/Reptilia	Chordata	Oil extracted is used for body massage, and also to enhance sexual power in humans.
62	***Vanellus indicus***/Tateeri, Red watteled lapwing	Charadriidae/Aves	Chordata	It is an alarming bird against snakes, jackals, mongoose, cats etc.
63	***Varanus griseus***/Kroh,	Virinidae/Reptilia	Chordata	Oil obtained from it is used for massage of body and for sexual weakness. Its skin is also used for painting and art work.
64	***Vipera russelli***/Russel viper	Viperidae/Reptilia	Chordata	Captured and sold for anti-venome production.
65	***Wallago auto***/Malli	Siluridae/Teleostomi	Chordata	Used as food after cooking. Its oil is used in joint pain. Scales are used in paintings with glue and paint spray.

### Multinomial logit model estimation results

Multinomial logistic approach is a good tool in studying ethnobiological research where there is need to study multiple usages of different or/and same plants (and animals) all together. The comparisons can be made and the identification of the plants (and animals) is more important for medicinal use etc. It may be helpful in deciding to produce the medicinally important plants (and animals) at commercial level.

Multinomial logit model estimates the use of plants and animals. The maximum likelihood procedure has been used to maximize the log likelihood function after a number of iterations. The choice of plants (and animals) as medicine and food medicine is used as main cases. There is also food and medicine combined its part while all other uses of plants (animals) have been left out of the regression as the base case. The probability of choosing each use of plant (and animals) was assumed to be the function of their respective categories. The selected model for plants (and animals) is log likelihood function which is maximized at −95.737 (−44.82) after 19 (35) iterations. A Pseudo R2 for plant (and animals) model is 0.153 (0.210) and likelihood ratio of chi-square is also significant at less than one percent in both of the models. The model is significant according to the tests used in the analysis. Most of the individual coefficients are highly significant in both of the models. Some were found to be jointly significant. Positive (or negative) slope coefficients imply that probability of each selected plant (and animal) usage increases (or decreases) for the active plant (and animal) category (Tables 3 and 4).

**Table 3:** Multinomial logit statistics for plant use selection across different plants categories

**Variables**	**Medicinal use of plants**			**Food use of plants**			**Plants’ combined use of food & medicine**		
	**Coefficients**	**RRR**	**Marginal effects**	**Coefficients**	**RRR**	**Marginal effects**	**Coefficients**	**RRR**	**Marginal effects**
Arial = 1, Underground = 0	−20.1481	0.00	−0.0236	−0.1853*	0.831	0.1215*	−20.973*	0.00*	−0.4998*
Annual = 1, Perennial = 0	1.0036	2.728	−0.1342	1.754**	5.778**	0.0056	3.0207*	20.51*	0.5212*
Constant	18.7099			−1.9742*			19.0571*		
**Overall characteristics**									
No. of observations			91						
No. of iterations			19						
Pseudo R^2^			0.153						
LR Chi square			34.6*						
Log likelihood			−95.736						

Note: All other uses is taken as the base outcome. *,** shows the significance at 1 percent, 5 percent respectively.

**Table 4:** Multinomial logit statistics for animals use selection across different animals’ categories

**Variables**	**Medicinal use of animals**			**Food use of animals**			**Animals’ combined use of food & medicine**		
	**Coefficients**	**RRR**	**Marginal effects**	**Coefficients**	**RRR**	**Marginal effects**	**Coefficients**	**RRR**	**Marginal effects**
Domestic = 1, Wild = 0	−35.5876*	0.000	−0.087**	71.6471*		0.000	−1.1528	0.316	−0.1306
Verteberates = 1, Inverteberates = 0	−0.8245	0.439	−0.00003	−72.7121*		−0.000*	1.0320	2.807	0.1315
Herbivors = 1, Carnivors = 0	−1.4662	0.231	−0.00007	0.4495	1.568	0.000	1.7656	5.845	0.2212**
Constant	−0.2921			−36.669			−3.2930		
**Overall characteristics**									
No. of observations			64						
No. of iterations			35						
Pseudo R^2^			0.2101						
LR Chi square			10012						
Log likelihood			−44.82						

Note: All other uses is taken as the base outcome. *,** shows the significance at 1 percent, 5 percent respectively.

The dummy variable coefficient for aerial and underground plant parts is negative and significant for all uses of plants except the medicinal ones which is insignificant. However, marginal effect of medicinal usage of plants is positive in contrast to food usage of aerial parts of plants. It implies that underground plant parts are more likely to be used for medicine in contrast to aerial ones which are generally used for food purposes. It also reflects that aerial plant parts are less likely to be used for medicinal purposes than that of underground parts of the plant. These findings are consistent with the results as given in [9]. The binary covariate annual coefficients and perennial are significant and positive for each use of plants except medicinal application where the coefficient is positive but not statistically significant. Marginal effects for these plants are non-significantly negative for medicinal plants usage and positive for the domain of food purposes. This shows that annual plants are less likely to be medicinally used as compared to the perennial plants. This finding is also consistent with the results of [9]. However, the positive marginal effects for food plants reveal that they are more likely to be used for food as compared to the perennial plants but none of the significant values was as small (0.0056) as can be effective in food use. Marginal effect for combined usage of food and medicine is statistically highly significant but negative for dummy variable of aerial and underground plant parts. This is positive for dummy variable of annual and perennial plants that confirms the previous results (Table 3).

The domestic and wild categories of dummy variable have negative significant coefficients for medicinal use of plants while it has negative non-significant coefficients of combined use of food and medicinal use of plants. It has positive significant coefficient for food use of plants. The significant negative marginal effect explains that wild animals are more likely to be used for medicinal purposes than the domestic animals. However, there is very low insignificant value of marginal effect of food use of animal which approaches to zero. It shows no difference in (equal use of) domestic or wild animals. Marginal effects of vertebrate and invertebrate animals are significant for food usage and non-significant for medicinal usage (value approaches to zero). However, the marginal effect for their combined use was found to be positive but insignificant. It implies that vertebrates are less likely to be used in medicine and food usages as compared to invertebrates while the difference is negligible. Likewise, the coefficients of the dummy variable for the herbivores and carnivores are negative for medicinal usage of animals and positive for their food use. Both of them are insignificant but their combined usage has positively significant coefficient which implies that herbivores are more likely to be used as medicinal and food purposes as compared to that of carnivores (Table 4).

## Conclusion

This paper has reported ethnobiology of 91 plants and 61 animals of important species that are statistically verified. These species are frequently used by the natives especially for medicinal purposes. The collected ethnobiological data may provide basis to formulate a policy for biodiversity conservation and community development. Therefore, it is articulated that such ethnobiological studies can make significant contributions to indigenous knowledge as well as to the sources of raw materials for the development of commercial pharmaceuticals and neutraceuticals. The native biota of Kala Chitta hills is threatened by factors such as extensive fuel wood consumption, hunting of wild animals, grazing, expansion of new agricultural lands, buildings, roads and unsustainable picking of plants to generate income. Punitive measures should be taken to ensure the inclusion of relevant flora and fauna within conservation designations.

## Competing interests

The authors declare that they have no competing interests.

## Authors’ contributions

All authors have significant contribution to design field study, data collection, analysis and final manuscript write-up. All authors have gone through this paper and approved the final manuscript.
